# The biography of the immune system and the control of cancer: from St Peregrine to contemporary vaccination strategies

**DOI:** 10.1186/1471-2407-14-595

**Published:** 2014-08-16

**Authors:** Bernd Krone, Klaus F Kölmel, John M Grange

**Affiliations:** Institute of Virology of Georg August University Göttingen, Göttingen, Germany; Medical Laboratory, Kurt-Reuber-Haus, Herkulesstraße 34a, 34119 Kassel, Germany; Dermatologic Clinic of Georg August University Göttingen, Göttingen, Germany; London Clinic Cancer Centre B2, 22 Devonshire Place, London, W1G 6JA UK

**Keywords:** Leukaemia, Melanoma, Endogenous retroviruses, Yellow fever vaccine, Bacille Calmette-Guérin

## Abstract

**Background:**

The historical basis and contemporary evidence for the use of immune strategies for prevention of malignancies are reviewed. Emphasis is focussed on the Febrile Infections and Melanoma (FEBIM) study on melanoma and on malignancies that seem to be related to an overexpression of human endogenous retrovirus K (HERV-K).

**Discussion:**

It is claimed that, as a result of recent observational studies, measures for prevention of some malignancies such as melanoma and certain forms of leukaemia are already at hand: vaccination with Bacille Calmette-Guérin (BCG) of new-borns and vaccination with the yellow fever 17D (YFV) vaccine of adults. While the evidence of their benefit for prevention of malignancies requires substantiation, the observations that vaccinations with BCG and/or vaccinia early in life improved the outcome of patients after surgical therapy of melanoma are of practical relevance as the survival advantage conferred by prior vaccination is greater than any contemporary adjuvant therapy.

**Summary:**

The reviewed findings open a debate as to whether controlled vaccination studies should be conducted in patients and/or regions for whom/where they are needed most urgently. A study proposal is made and discussed. If protection is confirmed, the development of novel recombinant vaccines with wider ranges of protection based, most likely, on BCG, YFV or vaccinia, could be attempted.

**Electronic supplementary material:**

The online version of this article (doi:10.1186/1471-2407-14-595) contains supplementary material, which is available to authorized users.

## Background

**Figure 1:**
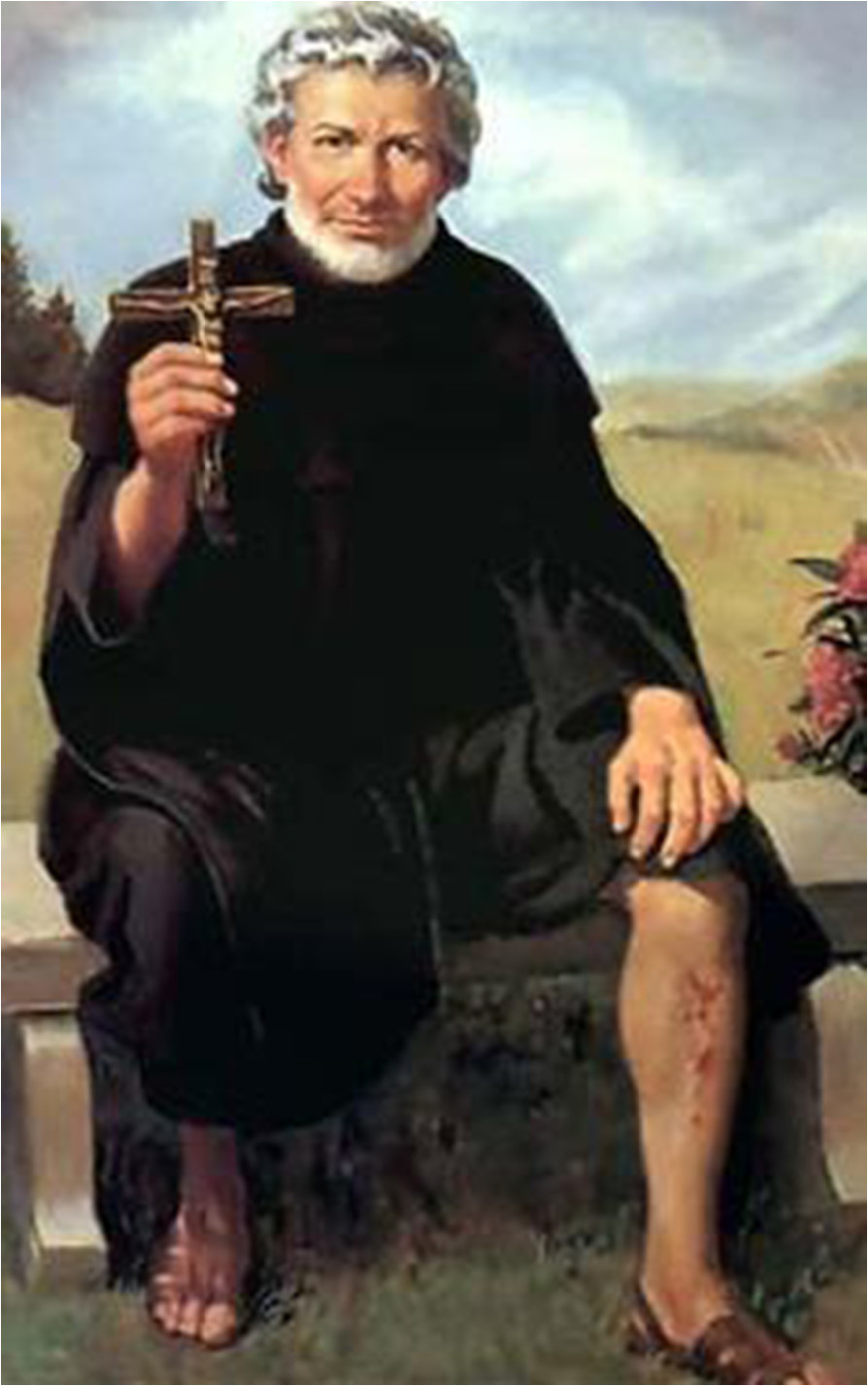
**St. Peregrine** **(1260–** **1345).**

As a young man, Peregrine Laziosi (1260–1345, Figure 1) developed a large swelling on a leg (accounts differ as to which leg), which was diagnosed as cancer. The lesion ulcerated and the stench – a sure sign of infection – was said to be so overpowering that his friends could not bear to stay with him for long. Amputation seemed the only option but, when the surgeons came to operate, the tumour was found to be in regression and it eventually healed completely. He had no recurrence of the cancer, lived to be 85 years of age, was canonized as Saint Peregrine in 1726 and is recognized by the Roman Catholic Church as the Patron Saint of cancer patients [1]. This is just one example of reports, over past centuries, of the spontaneous remission and even complete resolution of cancers following some form of infection [2–9].

In 1875 Campbell de Morgan, a surgeon at the Middlesex Hospital in London, reported that regressions and remissions of cancers sometimes occurred after post-operative infections, particularly the streptococcal infection erysipelas [10]. De Morgan wrote, “this is an occasional event which is very important as it encourages us to hope that a cure may yet be found for the disease.” In the light of recent advances in the immunology of cancer the time may well be approaching when an elucidation of the mechanisms underlying this ‘occasional event’ could lead to advances in the prevention and therapy of this widespread disease.

Campbell de Morgan’s observation that remissions sometimes occurred after post-operative streptococcal infections inspired some workers to undertake the risky procedure of deliberately inducing erysipelas in cancer patients. Subsequently, an American surgeon, William Coley, developed bacteria-free extracts of streptococci and other bacteria (“Coley toxins”) and reported their successful use in the therapy of cancers, especially sarcomas, between 1881 and 1936 [11–13]. Unfortunately Coley, a mild mannered and unassuming gentleman, did not adhere to rigorous scientific protocols in his studies and he was marginalized by forceful personalities advocating radiotherapy. Notwithstanding, an analysis of his results with cancer deemed inoperable undertaken in 1994 revealed a remission rate of 64% and a five-year survival rate of 44%, results equal to or better than those with modern therapies [14]. There have been several more studies on this topic [15–23], but the evidence for the effectiveness of this therapeutic approach remains disputed.

The implication of postoperative infections for the prognosis of cancer patients has been investigated in numerous comparative studies, some of which demonstrated a better prognosis for patients who had a postoperative infection compared to patients without infections [24–33]. A recent study, for example, on the effect of post-operative infection on outcome after surgery for osteosarcoma showed that the 10-year survival among those who developed deep tissue infection within one year of surgery was 84.5%, compared to 62.3% in those who did not develop infections (p = 0.017) [34]. Many of the earlier studies did, however, have severe methodological flaws and the results were quite heterogeneous and contradictory.

There has, in recent years, been a great upsurge of interest in the immunology of cancer and it has become clear that tumours are heterogeneous structures that, during their development and growth, become ‘sculpted’ or ‘edited’ by immune responses and, as a result, pass through the ‘three E’s’ of elimination, equilibration and escape [35]. Even when a tumour is large enough to present clinically, the immunoediting continues in a Darwinian fashion with selection of cells expressing novel antigens which avoid recognition by the induced immune responses [36], explaining the short-lived effects of immunotherapeutic strategies based on single, or a few, tumour antigens.

It is also now appreciated that chronic inflammation is an essential element of cancers and it has indeed been termed ‘the other half of the tumour’ [37]. The normal healing process relies on inflammation, collagen production, angiogenesis and cell proliferation and, in a description of the similarities between tumour stroma formation and wound healing, tumours have been referred to as “wounds that do not heal” [38], while in 1972 Sir Alexander Haddow suggested that tumour growth is the result of overhealing [39]. In addition, chronic inflammation has been linked to the generation of local and general patterns of immune suppression that protect tumours from immune recognition and attack [40].

Numerous attempts have been made in recent years to develop immunotherapeutic procedures for established cancers, though of greatly varying efficacy and cost. Much less work has been conducted on preventive immune strategies and, with the notable exception of human papilloma virus vaccine for the prevention of cervical cancer, no vaccines specifically for the prevention of cancer are in routine use. The subject of this review, however, is the possible use of available vaccines developed for the prevention of common infectious diseases to reduce the risk of at least some cancers.

### Infections and cancer

The relationship between infection, and associated inflammation, and cancer is a complex and paradoxical one and there are several well described examples of cancer being the direct consequence of infection [41]. Around 2 million of the 12.7 million new cancer cases worldwide in 2008 (16.1%) were assumed to be related to infection, principally *Helicobacter pylori*, hepatitis viruses, and the human papilloma virus, with a higher proportion in developing countries (22.9%) than in developed ones (7.4%) [42]. The large majority of cases of cancer, especially those in the developed nations, are therefore not caused by infection – on the contrary, there is growing evidence that a history of certain infections and environmental exposure to certain populations of micro-organisms, as well as some types of vaccination, may induce patterns of immune reactivity that reduce the risk of at least some cancers. It has, for example, been claimed that while certain chronic infections predispose to cancer, acute infections are antagonistic [43], and that life-style factors leading to exposure of infants to acute infections, such as attendance at day-care units, lowers the risk of acute lymphoblastic leukaemia and melanoma [44, 45]. Similar observations on acute lymphoblastic leukaemia in childhood had been made previously in a case–control study [46].

A study of an adult population in Italy demonstrated an association between a history of common childhood infectious diseases (measles, chickenpox, rubella, mumps and pertussis) and the risk of developing chronic lymphatic leukaemia (CLL), with a strong inverse relationship between the risk of CLL and the number of infections (p = 0.002) [47]. In view of this cumulative protection from different infectious agents, the authors concluded that an explanation based on the ‘hygiene hypothesis’ was more likely than an anti-cancer effect of the viruses. Despite a wealth of epidemiological studies on infections and cancer risk the case seems, however, to be unresolved.

Another, though possibly related, example of an apparent protective environmental effect on cancer is provided by the association of exposure to cattle in the dairy farming industry that apparently protects against several types of cancer, with statistically significant associations for lung, bladder, pancreatic, and oesophageal cancer [48]. The degree of apparent protection is related to the intensity of the exposure (the number of cattle to which the farmers were exposed) but wanes when the farmers change to other occupations. These findings were confirmed in a high-quality study in Finland [49], and in a meta-analysis of published reports [50]. Although it has been claimed that the agents conferring protection are endotoxins that are present in the dust derived from cattle faeces [51], it is equally likely that apparent protection is mediated by various genera and species of actinomycetes, which are likewise present, and at high densities, in cowsheds. There were also earlier investigations suggesting that persons who are exposed to endotoxins in other occupations likewise have a lower cancer risk [52].

### ‘Darwinian medicine’

The risk of developing cancer is rising globally, especially in the industrially developed nations where only one sixth of the human population reside, yet almost half the cases of cancer occur [53]. While cancer incidence rates are mostly higher in developed as compared with developing countries, the latter show a higher secular increase in incident rates. This rise has been attributed to an increasing portion of the population reaching a more advanced age but, as an increase is also seen in cancers affecting younger people such as melanoma [54, 55], this may be only part of the explanation. The rising trend commenced early in the 20^th^ century, coinciding with the massive recession of the major plagues, notably smallpox and tuberculosis, as well as of other serious infectious diseases [56]. The roles that infections seem to exert on cancer and cancer risk can be either beneficial or detrimental and, since there is clearly an involvement of other environmental and genetic factors a weakness of many studies is that corrections for the influence of these factors were not made so that the role of infections is still unresolved.

Moreover, there has also been an increase in the incidence of several classes of disease associated with chronic inflammation in the developed nations. These include asthma, allergic disorders, vasculitis, neurodegenerative disorders including multiple sclerosis, autoimmune diseases such as type-1 diabetes and inflammatory bowel disease [57]. It is noteworthy that all these disorders are associated with chronic inflammation attributable to dysregulated immune responses [58]. The immunological anomalies underlying these diseases may also be involved in at least some forms of cancer [59], although it remains to be determined whether such inflammation contributes to the incidence of cancer or whether it is just a common epiphenomenon.

From the moment of birth and even, though less directly, from the moment of conception, a human being is exposed to a vast range of members of the microbial universe. It has indeed been estimated that for every human cell in the body there are around ten micro-organisms dwelling particularly in the intestine, the upper respiratory tract and the skin. It is now generally appreciated that, without underestimating the role of genetic factors, this microbial population, the microbiome [60, 61], as well as more transient infecting agents, play a crucial role in driving the maturation of the immune system and the generation of complex immune regulatory networks [62].

Human beings have evolved to ‘expect’ immunologically effective contact with certain classes of micro-organisms, including commensals and parasites, and exposure to at least some of them seems to be required for the development of a well-regulated immune system [63]. Some experimental studies in animals gave indications that some parasites might have a potential to reduce the risk of cancer [64–66]. Unfortunately, many hygiene-related factors in the industrialized nations prevent adequate exposure to these micro-organisms which have been termed our ‘Old Friends’ [63]. Indeed, this issue is receiving much attention within the emerging discipline of Darwinian Medicine [62, 63].

Another consequence of improved standards of hygiene is a change in the sequence in which infections by micro-organisms occurs. An early infection by a given micro-organism will elicit immune responses principally to its dominant epitopes, but if the infection occurs subsequent to infection by other micro-organisms bearing cross-reactive epitopes, the response may well be directed principally towards alternative, non-dominant, epitopes with quite different consequences for the host. The phenomenon has been termed ‘Original Antigenic Sin’ [67], and we have previously postulated that the increased incidence of multiple sclerosis in the developed nations over the last 150 years is a consequence of an altered immune reactivity to the Epstein-Barr virus, a strong risk factor for the disease when it is acquired at a later period in life (late teens or early adult life) rather than in infancy [59, 68]. The immune system of each individual therefore develops its distinctive ‘biography’ so that the response to a given antigenic challenge may vary from one individual to another with, for example, generation of regulatory T-cells in one individual and proliferation of various effector T cells in another [68]. This raises the question of whether the ‘biography’, especially if affected by hygiene-related factors, is also a risk-determining factor for cancer and whether this risk can be reduced by altering the biography by, for example, appropriate vaccination strategies.

### The case of vaccination with Bacille Calmette-Guérin (BCG) and cancer risk

Naturally occurring infections and environmental exposures to microbial populations provide, by their very nature, highly unpredictable ways of preventing cancer whereas vaccinations provide in principle a much more rational and safer means to achieve this aim. Likewise, despite the observations of Coley and others, therapeutic and/or preventive vaccines that do not induce fever would be far more acceptable to both regulatory authorities and patients.

BCG vaccine, a living and attenuated derivative of *Mycobacterium bovis*, has been used, though with very variable results, for the prevention of tuberculosis for around 90 years. Being a whole bacterium with an extremely complex adjuvant-rich cell wall it would be surprising if it did not have effects on the human immune system beyond inducing immune responses directed at the tubercle bacillus. Indeed, commencing in 1935 [69], numerous attempts have been made to use BCG as an immunotherapeutic agent for treatment of cancer though, with the notable exception of superficial bladder cancer, with very variable and generally disappointing results [70].

By contrast, there have been several reports that BCG vaccination affords a useful degree of protection against leukaemia and certain other malignancies in children. The early reports, commencing in 1970, generated considerable controversy with conflicting data being reported by different workers. When, however, the data from all published reports were compared, it became apparent that protection against leukaemia was conferred in those settings in which BCG was administered very early in life and/or where vaccination conferred significant protection against tuberculosis [71].

In Finland it was shown that a positive tuberculin reaction indicative of infection by *Mycobacterium tuberculosis* or vaccination with BCG led to a reduced risk of all types of leukaemia over a 30 year follow-up period, with natural infection and BCG vaccination conferring equal levels of protection [72]. In this context it is possible that natural infection by *Mycobacterium bovis* (from which BCG was derived) protected against leukaemia as a 4.5% annual increase in rates of this disease in Great Britain between 1911 and 1959 has been reported [73]. Notably, 1911 was the year that bovine tuberculosis eradication measures commenced in Great Britain and led to a reduction in viable bovine tubercle bacilli in cows’ milk [74], although of course there may be alternative explanations for the rise in the incidence of leukaemia.

There is considerable geographical variation in the protective efficacy of BCG vaccination against tuberculosis, ranging from around 80% to no protection, and even an increase of disease risk in some regions [75]. A widely accepted explanation for this variation is that environmental factors, notably exposure to populations of saprophytic mycobacteria in the water and soil, are able to prime the immune system to an inappropriate, Th2 polarized, pattern of reactivity that BCG is unable to reverse and may even boost [76, 77].

### ‘Failed immune stimulation’ as a melanoma risk factor and its interplay with other risk factors

There are a number of established melanoma risk factors, namely light skin pigmentation, intermittent sun exposure and multiple naevi, with some other candidate factors which are currently being investigated, such as exposure to heavy metals, polychlorinated biphenyls, pesticides, and genetic factors modifying response to environmental factors [78]. The potential protective effect of infections and vaccinations on cancer and cancer risk and progression of melanoma has, until recently, been largely neglected.

In the 1990s Kölmel and colleagues established a working group – Febrile Infections and Melanoma (FEBIM) – within the European Organization for Research and Treatment of Cancer (EORTC). Based on a pilot study [79] this group undertook a series of studies to establish the relationship between the risk for developing melanoma and a history of, initially, infectious diseases [80], and, subsequently, also of vaccinations [81, 82]. The study cohort included 603 cases and 627 population controls matched with respect to sex, age, and ethnic origin within each of a total of 11 study centres in six European countries and in Israel. The recruitment period for the investigation lasted from 1994 to 1997. The study investigated the effect of febrile infections and of certain vaccinations on the risk to develop melanoma as well as the duration of a risk reducing effect of the vaccinations and on possible synergistic, cumulative or non-cumulative effects of vaccinations and infections. Established melanoma risk factors (see above) were also determined and adjustments were made for these factors as well as for gender and age.

In the first report of the FEBIM group a significant level of protection against melanoma in those with a history of certain severe infections (sepsis, *Staph. aureus* infection, pneumonia, pulmonary tuberculosis) with fever of over 38.5°C was demonstrated [80]. It should, however, be noted that these apparently melanoma-protective infectious diseases have become rare in the industrialized nations. A subsequent study report included the history of vaccinations and demonstrated protection with an odds ratio of risk of 0.4 (95% confidence interval 0.18-0.85) in those vaccinated with BCG alone, 0.6 (95% CI 0.36-0.99) in those vaccinated with vaccinia alone and 0.41 (95% CI 0.25-0.67) in those receiving both vaccines [81]. The vaccines were both administered early in life and were associated with a long-lasting relatively strong protective effect against melanoma in the age group < 50 years with a waning of protection in older subjects (age group ≥ 50 years) but with a cumulative protective effect in those receiving both vaccines [81] (Table 1). Joint analyses of vaccinations and a history of serious infectious diseases (s) likewise exhibited a cumulative effect of the weaker protections [83] (Table 2).

**Table 1:** **Joint effects of vaccination with BCG and vaccinia on melanoma risk** ^**a**^

Co-variable	Only BCG	Only vaccinia	Both
*Age group*			
<50 years	0.23 (0.05-0.91) n = 25	0.31 (0.07-0.98) n = 111	0.27 (0.09-0.80) n = 299
≥50 years	0.74 (0.25-2.28) n = 20	0.69 (0.38-1.22) n = 362	0.48 (0.26-0.86) n = 313

^a^With respect to two age groups, **<**50 and **≥**50 years. Data expressed as Odds Ratios (95% Confidence interval), adjusted for centre, sex, ethnic origin, skin type, freckling index, number of naevi and number of sunburns. n = number of cases and controls. Summarized from the FEBIM study [81].

**Table 2:** **Joint effects of vaccination with BCG and**/**or vaccinia with history of serious infectious disease on melanoma risk**^**a**^

Co-variable *serious infectious disease*	Vaccination with BCG and vaccinia		Concomitant effect (type of)
	Yes	No	
≥1	1.00 n = 96	1.21 ((0.67-2.06) n = 12	
0	1.12 (0.30-4.30) n = 516	3.03 (1.58-5.97) n = 88	Cumulative
	Vaccination with BCG or vaccinia		
Serious infectious disease			
	Yes	No	
≥1	1.00 n = 98	1.97 (1.14-3.56) n = 12	
0	1.28 (0.35-4.90) n = 420	3.45 (1.79-6.80) n = 89	Cumulative

^a^Data expressed as Odds Ratios (95% Confidence intervals), adjusted for centre, sex, age, ethnic origin, skin type, freckling index, number of naevi and number of sunburns. n = number of cases and controls. Summarized from the FEBIM study [82, 83].

**Table 3:** **Joint analyses of the melanoma risk indicator** ’**not being vaccinated with either BCG or vaccinia**’ **and four co**-**variables of melanoma risk**^**a**^

Co-variable	Vaccination		Concomitant effect (type of)
	Yes	No	
*Skin*-*type* (*Fitzpatrick*)			
III/IV	1.00 n = 607	1.68 (0.93-3.05) n = 58	
II	1.80 (1.18-2.78) n = 396	2.15 (0.65-8.34) n = 30	
I	1.54 (1.16-2.05) n = 126	6.37 (3.50-19.64) n = 12	Synergistic
*Sunburns in life*			
0	1.00 n = 338	1.51 (0.76-3.01) n = 46	
1-5	0.93 (0.68-1.26) n = 596	2.29 (1.14-4.76) n = 45	
>5	1.39 (0.91-2.14) n = 191	5.30 (0.87-102.48) n = 8	Synergistic
*Naevi*			
0	1.00 n = 272	2.51 (1.16-5.77) n = 35	
1-4	1.05 (0.70-1.48) n = 387	5.24 (1.89-17.10) n = 22	
>4	1.56 (1.10-2.22) n = 456	1.71 (0.84-3.57) n = 42	Non-cumulative
*Freckles on arm*			
0	1.00 N = 457	3.26 (1.60-6.87) n = 39	
10-20	1.57 (1.17-2.10) n = 426	2.38 (1.09-5.32) n = 31	
>20	3.03 (2.13-4.35) n = 247	4.06 (1.78-10.17) n = 30	Non-cumulative

^a^Compared with ‘vaccinated with BCG and**/**or vaccinia’. Data expressed as Odds Ratios (95% Confidence Intervals) for melanoma risk, adjusted for centre, sex, age, and other known risk factors. n = number of cases and controls. Summarized from the FEBIM study [83].

Joint analyses of ‘vaccinated or not vaccinated with BCG and/or vaccinia’ (Table 3) with established melanoma risk factors showed synergisms or, in other words, indicated a substantial potential of these vaccinations to neutralize – at least in part – one or two major environmental melanoma risks indicated by skin type (according to Fitzpatrick) and number of sunburns in life, respectively, representing vulnerability of the skin by ultraviolet light and injury caused by it [83].

The relation of ‘vaccinated or not vaccinated with BCG and/or vaccinia’ with indicators of genetic melanoma risk, as indicated by number of naevi and number of freckles, respectively, was less straightforward. However, comparison of the reference of the supposed genetic risk indicators with the highest categories in the joint analyses led to the categorization as ‘non-cumulative’. This was a rather surprising observation.

### Interpretations of the FEBIM study

The FEBIM study led to the conclusion that vaccination with BCG and/or vaccinia over-rides a major genetic risk factor such as the expression of an oncogene involved in the pathogenesis of melanoma.

In principle all the findings of the study could be explained in at least two different ways. First, these vaccines may substitute for natural contact with micro-organisms for inducing regulatory mechanisms in the immune system [82, 83]. Secondly, the vaccines may generate cross-reacting immune responses directed on one or more epitopes expressed on potential precursor cells of many or all melanomas and eventually also on malignantly transformed melanoma cells. These two explanations are not mutually exclusive.

In support of the second explanation, a search of gene data bases by use of the Basic Local Alignment Search Tool (BLAST) showed that the relevant pathogens and vaccines (BCG and vaccinia) with a demonstrable protective effect, but not pathogens and vaccines not associated with protection, have epitopes homologous with HERV-K-MEL, an epitope encoded by a human endogenous retrovirus of the K series (HERV-K) [83, 84]. This epitope is expressed in the majority of melanomas, as well as on cells of atypical naevi (presumed potential precursors of melanoma), and to a lesser extent in certain other cancers, and is capable of generating CD8^+^ T-cells directed towards potential precursor cells of melanoma [84]. In this context there is evidence that expression of HERV-K in melanocytes can result in malignant transformation [85], and a mechanism could be the generation of an abnormal melanin capable of inducing harmful long living reactive oxygen species which may also have relevance to other disease processes, such as multiple sclerosis in which HERV expression occurs [86]. It should be noted that the putative oncogene is not the HERV-K-MEL peptide but the HERV-K-ENV protein, both being genetically encoded by the same gene complex though in different open reading frames.

The relevant vaccination(s) early in life could have generated expanded populations of specific T-cells cross-reactive with the HERV-K-MEL epitope. The situation has some analogy to the use of the human papilloma virus vaccine to prevent cervical cancer, except in the case of melanoma the target virus is not a replicating one and is endogenous rather than exogenous. It must also be emphasized that here (in case of the supposed inducible melanoma immune surveillance) endogenous and exogenous risks are merging into one and it remains a question whether and how they could be separated clearly from each other.

As, however, vaccinia vaccination is no longer used (vaccination of the general populations ceased around 1975) and neonatal BCG vaccination is currently restricted to certain ethnic groups and localities (although the upsurge of extreme resistant tuberculosis may lead to its more extensive use in the future) an alternative cheap and safe vaccine would be preferable for the prevention of melanoma. The same analysis used to identify the homologous epitopes to HERV-K-MEL in vaccinia and BCG vaccines also revealed a homologous epitope, with similar anchor sequences for HLA presentation, in the 17D yellow fever vaccine (YFV) [83]. Moreover, the induction of an anti-melanoma immune response was observed in Rhesus macaques vaccinated with YFV [87].

### HERV-related malignancies, ‘self-specific immunity’ and a mouse-melanoma model

In human beings the endogenous retroviral ENV gene [88], and the spatially closely related MEL gene [84], of HERV-K (the latter coding only for a peptide) can be expressed in human cells but, in contrast to the melanoma cells and their presumed precursors, are typically not found in normal cells. Anchor sequences of the hypothetical MEL peptide should facilitate its presentation, in particular by the HLA-A2 molecule that is present in about 70% of the European population. Besides melanoma, chronic lymphatic leukaemia, lymphomas and breast cancer express HERV-K antigens more frequently than healthy tissues [84, 89–91], and thus require consideration in this debate.

Current concepts in immunology indicate that vaccination does not just evoke immune responses to exogenous pathogens but can elicit immune responses and induce immune regulatory networks affecting endogenous (self-specific) epitopes [92–94]. In this context, the murine colorectal carcinoma CT26 and melanoma B16 express, respectively, products of the endogenous retroviral genes gp70 and p15E which are recognized by T cells and when animals with lung metastases due to these tumours were inoculated with dendritic cells pulsed with these endogenous antigens significant tumour inhibition was observed [95]. It must, however, be acknowledged that while vaccinia, BCG and the experimental mouse vaccines seem to reduce the risk of developing melanoma they have little or no potential for therapy of established tumours.

### Postulated critical aspects of cancer protective immunity inducible by vaccination

The ultimate question as to how immune reactivity prevents certain cancers has not yet been answered but several pointers to the answer have become apparent and merit further study. In this context it is noteworthy that prevention of malignant diseases, in particular of melanoma, seems to be achieved much more easily than the control of established disease by immunotherapy [96]. Moreover, prevention of melanoma by prior vaccination with BCG, vaccinia and/or YFV appears likely to rely on processes operating at a time *before* immune tolerance is induced. Establishment of immune tolerance in the tumour micro-environment is an essential element of tumour survival and progression and, once established, it is difficult to break [97, 98]. The attrition of local and of general immunity has been linked to chronic inflammation, and immune suppression is mediated by myeloid-derived suppressor cells, type-2 cytokine expression and alternatively activated, M2, macrophages that protect tumours from immune recognition and attack [40].

Furthermore, with respect to the ‘biography’ of the immune system, there is increasing evidence that cancer is associated with the nature and function of regulatory and helper T-cells, including a local and more general Th1 to Th2 shift [67, 68, 99]. Indeed, the characterisation of the types of tumour-infiltrating T-cells and macrophages appears to provide a clearer prognostic indicator than the conventional classifications based on extent and spread of disease [100].

A subset of CD8^+^ T-cells, the CD8 (+) CD44 (high) cells, are self-specific and appear to play a unique role in surveillance of host cells that have been altered by infection or malignant transformation [93]. Although in experimental settings these cells can be transformed by cytokines such as interleukin-2 to operate in a cytotoxic mode, it is uncertain whether a lasting therapeutic effect can become induced. For prevention it is more likely that another effector mechanism is involved; one that is not cytotoxic but suppresses the genetic expression of a gene such as the HERV-K-ENV postulated to be involved in oncogenesis [83, 99]. This might be described as a kind of immune repair operating at the time around tumour initiation. The self-specific immunity may not just be mediated by the specific CD8^+^ T-cells mentioned above but also by a subset of gangliosides, in particular some of the neo-lacto series, that are shed by interacting macrophages directly to the target cells [101–103].

### The ‘biography’ of the immune system and control of cancer

Immune memory in respect to protection against melanoma resulting from vaccinations early in life is clearly long-lasting. Vaccination with vaccinia and/or with BCG in early childhood resulted in a strongly reduced risk of melanoma in the age-group up to 50 years (OR = 0.27, 95% CI: 0.09-0.80) and the protection extended, though with reduced strength, beyond the age of 50 years (OR = 0.48, 95% CI: 0.26-0.86) [81].

Around 95% of the European population was vaccinated with vaccinia until about 1975 when it was terminated and around 50% received BCG vaccine until about 1990 when it was phased out except in certain high-risk groups in some countries. If the findings of the FEBIM study are correct, the consequence of these changes in vaccination strategies could be a continued rise in the incidence and risk of melanoma. The risk may be further enhanced by a reduction in the background protection induced by declined exposure to infectious and environmental agents.

In the past, the major plagues may indeed have been important as inducers of melanoma-preventing immune responses among the survivors. Homologies to the HERV-K-MEL sequence, the postulated target of the induced immune responses, were also found to be present in the causative agents of all the plagues that declined markedly in prevalence in the late 19th or early 20th century; namely, yellow fever, cholera, smallpox, typhoid, typhus, diphtheria, syphilis, tuberculosis and scarlet fever. Not all strains of streptococci express the HERV-K-MEL epitope [83], possibly explaining why Coley’s therapy with *Streptococcus pyogenes* was rather variable in effect until he added *Serratia marcescens*, which has an epitope with good homology with HERV-K-MEL. In this context, a case of regression of an advanced metastatic melanoma following diphtheria-pertussis-tetanus vaccination has been described and, in a discussion of possible mechanisms it was observed that homologous peptides to HERV-K-MEL are present in the three components of this vaccine [104].

### A possible melanoma protective effect of vaccination with yellow fever 17D

A study was undertaken in the Veneto, Northern Italy, where the administration of the yellow fever 17D vaccine is strictly documented, to determine whether this vaccine might likewise confer protection against melanoma [105]. As, in general, only those intending to travel to tropical countries are vaccinated against yellow fever, confounding factors are introduced as there are socio-economic differences between vaccinated and unvaccinated groups which could affect the risk of melanoma [106].

Accordingly, the analysis was performed within the vaccinated group (n = 28,306) by comparing melanoma with cancers not expressing the HERV-K-MEL epitope and this revealed a significant reduction in the risk of melanoma among those vaccinated 10 or more years previously [105]. The odds ratios and 95% confidence intervals (CI) were calculated by means of logistic regression models. Odds ratios in the 0–4, 5–9 and ≥ 10 year time since vaccination (TSV) groups were, respectively, 1.00, 0.96 (CI 0.29–1.67) and 0.26 (CI 0.07-0.96). In an interim report from an ongoing follow-up of the Italian cohort (n = 27,905 vaccinees, 401 were not traced) the follow-up time was extended from 31 December 2001 to 31 December 2005 [107]. Person-years (PY) were broken down to five-year classes of age, gender, and TSV. The percentage of PY between 18 and 64.9 years of age was 93% in the cohort and 68% in the general Veneto population in 1996 (mid year of the observation period 1987–2005), while the percentage of population aged 65+ years were 7% and 17% in the cohort and Veneto population, respectively. Within the cohort, the percentages of males and females were similar both before 65 years of age (93% vs. 93%) and after 65 years of age (7% vs. 7%). Moreover, the percentages of PY above 65 years of age were 6% and 12%, respectively, in the first and second class of TSV. Therefore PY tend to increase with TSV and the two variables are correlated with each other.

The record-linkage with VTR data returned 57 cases of melanoma (37 in the initial study) and (used as the control group) 1214 other site cancers (except skin cancers), an overall of 1271 cases (830 in the original study). TSV was broken down in two classes, <10 (n = 46 cases, 799 controls), and ≥ 10 years (N = 11 cases and 415 controls) [107]. The odds ratio and 95% confidence intervals were 1.00 (reference) and 0.48 (0.25-0.95), p = 0.035 and support the original observation. Incidence rate ratio (IRR) with 95% confidence intervals, calculated with Poisson regression, was 0.59 (0.30-1.16), p = 0.10.

Subsequently, an independent study on the protective effect of yellow fever vaccination on melanoma was conducted on subjects on active duty in the armed forces in the United States (US) who had received YFV or other vaccinations between January 1, 1999, and June 30, 2009 [108]. The study included 638 cases of melanoma and 6,372 healthy matched controls and showed a lowering of the risk of melanoma in the vaccinated TSV ≥10 year group, with an odds ratio of 0.70 (95% CI 0.29–1.67) but this was not statistically significant.

One advantage of the US study was that it involved a much more homogeneous population than that in the Italian study, thus removing many confounding factors and permitting a direct comparison of those vaccinated or not vaccinated with YFV. A weakness, however, was that the maximum time since vaccination (TSV) was only 11.5 years, with only 14.9% of subjects being in the TSV ≥ 10 year-group, whereas the maximum TSV in the Italian study was 22.6 years, and it was demonstrated that protection was only evident in the ≥10 year group. It is possible therefore that a follow-up study in the US might demonstrate a significant level of protection in the ≥10 year group.

The data from the Italian study indicate the need for further studies and if the results of this study reflect the real situation they give support to the concept that, in order to have a protective effect, vaccination (whether BCG, vaccinia or YFV) must be given at or before the time of the initiation of the malignant process, long before clinical manifestation of the disease which, in the case of melanoma is about 10 years [59, 105].

### Effect of prior vaccination on the clinical course of melanoma

**Figure 2:**
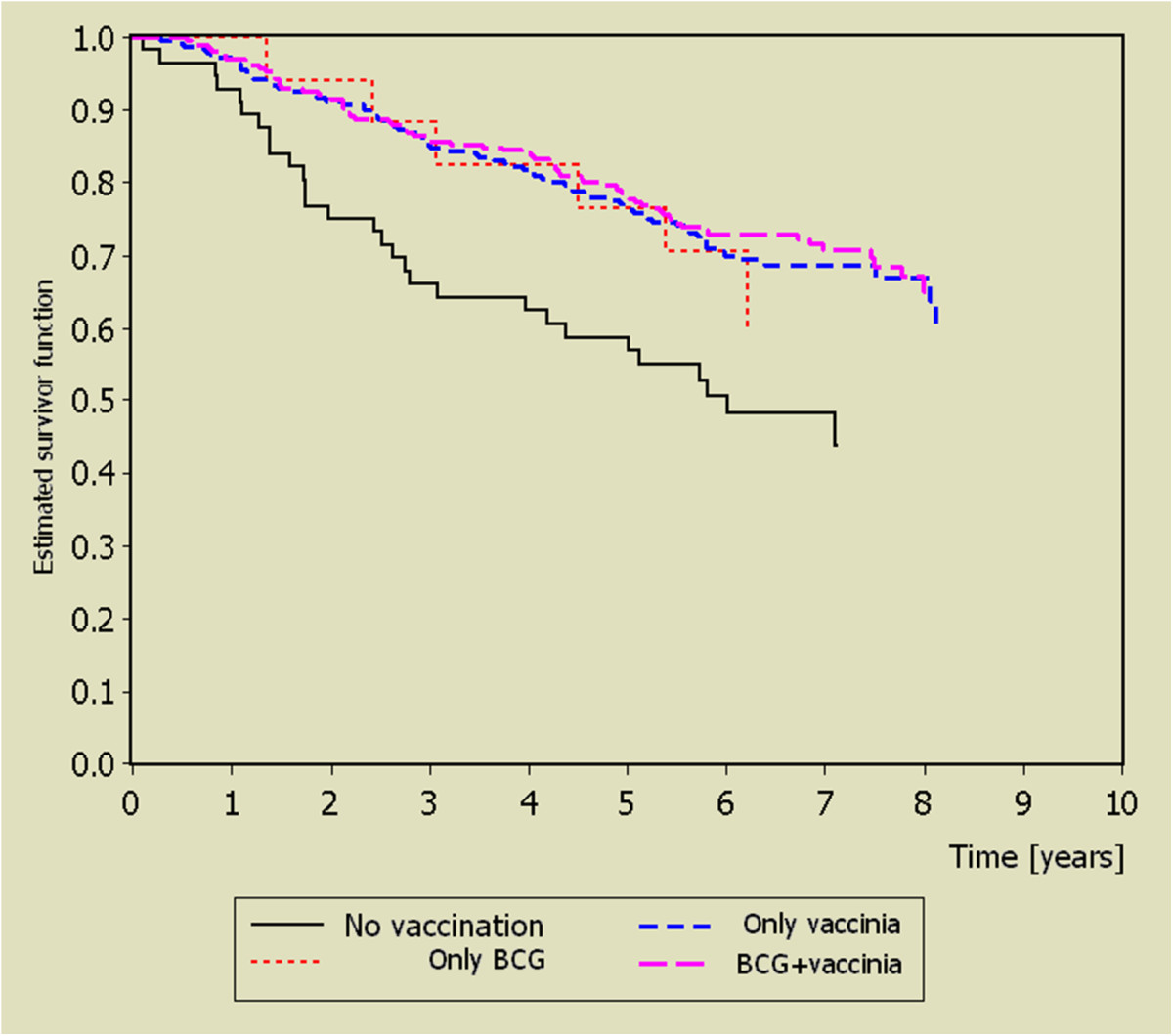
**Kaplan-** **Meier estimates for overall survival of melanoma patients enrolled immediately after excision of the primary tumour,** **from reference** **[**[109]**].**

As an important and unique extension of the FEBIM study, the effect of infections and vaccination with BCG and vaccinia on the progression of melanoma in those in whom the disease was not prevented was investigated (the prospective study arm) [109]. From the initially recruited 603 patients from 11 centers in six European countries and Israel 30 patients classified as having melanoma *in situ* were omitted from the follow-up and 31 (5%) of the patients were lost for the follow-up. Thus the outcome of 542 patients was evaluated. The survival of the patients, after surgery, who had been vaccinated with vaccinia, BCG or both was significantly better than that of the unvaccinated patients, as shown in Figure 2.Importantly, the differences shown in Figure 2 persisted after adjustment for several prognostic factors in a multivariate analysis. This is of importance for the appraisal of the study results, as otherwise it cannot be excluded that the differences between the unadjusted survival curves are merely due to bias from confounding factors. For example, social status may well be related to a willingness to participate in early detection programs, resulting in different tumour stages at the first diagnosis of melanoma.

This finding is without precedent. One possible explanation is that the development of a malignant disease such as melanoma is typically not the result of a single process but to an evolutionary progression involving several processes occurring at different stages of the disease. This can be understood in relation to the concept, discussed above, of ‘immunoediting’ as the tumour evolves through the stages of elimination, equilibration and escape, and which continues after clinical presentation [35]. Thus immune responses that are unable to prevent initial elimination and subsequent escape of a tumour from a state of equilibrium may nevertheless inhibit later events such as local invasiveness and the establishment of metastases.

Another possibility is suggested by the recent observation that expression of the HERV-K-ENV gene, being closely associated with the HERV-K-MEL, leads via the ENV peptide to the generation of atypical multinucleate cells which have growth and survival advantages, contributing to tumour progression [110]. Elimination or repair of cells expressing this peptide by CD8^+^ cytotoxic cells could therefore be advantageous to the patient.

## Discussion

Prior claims that BCG vaccination affords protection against certain malignancies have been reviewed, together with the results of the more recent FEBIM study. In the latter study, infections and a broader range of vaccinations, including vaccinia, which may have conferred protection, have been investigated and adjustments were made for other risk factors. Moreover, the subsequent studies on yellow fever vaccination [105, 107], gave a preliminary indication that YFV in adults might also contribute to such protection, although confirmative studies are required especially as a nested case approach and has to be handled with great caution [111].

On the other hand, there are advantages in features of the nested case approach as used addressing a possible tumour protective potential of yellow fever vaccination [105, 107]. The study design has advantages as it ensures that patients and controls are coming from the same source group of persons, thus minimizing diverse confounding factors such as those resulting from differences in the socio-economic status. Since, however, the controls also have various tumours, the relevance of the observed protection to the general population is uncertain [112]. Nevertheless, such studies could give answers to the following questions.

○ Does yellow fever vaccination afford protection against any other malignancies?

○ Is there indeed an association between expression of HERV-K genes within the tumours and protective efficacy of vaccination and, if so, is this a necessary or sufficient condition?

○ Could the risk of breast cancer, which often expresses HERV-K ENV [90], be prevented by BCG or yellow fever vaccination?

○ Is the time interval between vaccination and onset of protection the same for all malignancies?

○ What is an appropriate age for vaccination in order to achieve optimal benefit?

The latter also raises the question whether there is an age group of persons in specific need of such vaccination (s) while other age groups might have sufficient protection from alternative (environmental) immune contacts.

The yellow fever vaccine has many advantages over other currently available vaccines, as it is not only one of the most effective vaccines ever developed [113] but is cheap and relatively safe [114]. Indeed, in the 70 years or more elapsed since its development, YFV 17D has been administered to over 540 million people globally, with very rare (1 in 250,000) cases of serious adverse events and it can, in principle, induce protective immunity persisting up to 40 years [114], although re-vaccination is recommended after 10 years. Whereas BCG vaccination is most effective when given early in life (at least in respect to tuberculosis), YFV can be administered to adults, thereby addressing the age group most at risk of cancer.

Vaccinia vaccination is now principally of historical interest. However, since the vast majority of humans have been vaccinated in early childhood until about 1975 this is still to be considered in all studies investigating a possible protective effect against malignancies. The case of BCG and possibly of the newer mycobacterial vaccines undergoing evaluation is different. BCG in newborns is still in use in many tropical countries and newer non-replicating vaccines might find a use in adults. Therefore it is highly desirable that all future studies on risk factors for melanoma and other malignancies should investigate in parallel the status of vaccination with BCG, vaccinia and yellow fever.

The observation that BCG (and, historically, vaccinia) vaccination early in life improved the prognosis of patients after surgical therapy of melanoma is of practical importance as the prognosis of inoperable melanoma is poor. Further studies are required to determine whether BCG vaccination has a similar beneficial effect in other forms of cancer. We therefore open the debate as to whether extensive controlled vaccination studies should be undertaken in patients and/or regions for whom/where they are needed most urgently.

### Summary

The journey from Saint Peregrine’s ‘miraculous’ cure to contemporary vaccination strategies for the prevention or cure of malignant disease has been a long one and, unfortunately, it is supported so far mostly by observational studies as reviewed above rather than by mechanistic ones. Nevertheless, the more recent studies including those by the FEBIM group suggest that measures for prevention of some malignancies such as melanoma and some forms of leukaemia are already at hand: BCG vaccination of new-borns and (for melanoma) YFV of adults. We concede that the evidence of their benefit for prevention of malignancies needs to be strengthened by further studies and, as some other cancers also express HERV-K epitopes [84, 89, 90, 111, 115], these could likewise be the subjects of further studies. Such studies could well pave the way to the development of recombinant vaccines with improved and extended properties and these might well be based on YFV, mycobacterial and/or vaccinia vaccines.

## Authors’ information

BK, PhD, MD, worked for more than 7 years in the field of biomolecular chemistry and for 22 years in the field of virology. His recent interests include the role of endogenous retroviruses in carcinogenesis. KFK, MD, was engaged in the therapy of advanced melanoma, he was a pioneer in Germany in conducting campaigns for the public awareness of early recognition of melanoma and he led the FEBIM study on the impact of infections and vaccinations on the subsequent risk of melanoma. JMG, MD, MSc, has for many years worked extensively on the microbiology and immunology of chronic infection, especially tuberculosis and, since 1995, on the immunology and immunotherapy of cancer.

## Authors’ original submitted files for images

Below are the links to the authors’ original submitted files for images. Authors’ original file for figure 1 Authors’ original file for figure 2

## Abbreviations

BCG: Bacille Calmette-Guérin
CLL: Chronic lymphatic leukaemia
FEBIM: Febrile Infections and Melanoma working group
EORTC: European Organization for Research and Treatment of Cancer
HERV-K: Human endogenous retrovirus-K
PY: Person years
TSV: Time since vaccination
YFV: Yellow fever vaccine.

## References

[1] Jackson R (1974). Saint Peregrine, O.S.M. - the patron saint of cancer patients. Can Med Assoc J.

[2] Maurer S, Kölmel K (1998). Spontaneous regression of advanced malignant melanoma. Onkologie.

[3] Hobohm U (2001). Fever and cancer in perspective. Cancer Immunol Immunother.

[4] Hobohm U (2005). Fever therapy revisited. Br J Cancer.

[5] Rohdenburg GL (1919). Fluctuations in the growth energy of malignant tumors in man, with especial reference to spontaneous regression. Cancer Res.

[6] Everson TC, Cole WH (1956). Spontaneous regression of cancer: preliminary report. Ann Surg.

[7] Everson TC, Cole WH, Everson TC, Cole WH (1966). Spontaneous regression of malignant melanoma. Spontaneous regression of cancer.

[8] Stephenson HE, Delmez JA, Renden DI, Kimpton RS, Todd PC, Charron TL, Lindberg DA (1971). Host immunity and spontaneous regression of cancer evaluated by computerized data reduction study. Surg Gynecol Obstet.

[9] Nauts HC (1980). The apparently beneficial effects of bacterial infections on host resistance to cancer. Cancer Res Inst Monograph #8.

[10] Grange JM, Stanford JL, Stanford CA (2002). Campbell de Morgan’s ‘Observations on cancer’, and their relevance today. J R Soc Med.

[11] Hoption Cann SA, van Netten JP, van Netten C (2003). Dr William Coley and tumour regression: a place in history or in the future. Postgrad Med J.

[12] Jessy T (2011). Immunity over inability: The spontaneous regression of cancer. J Nat Sci Biol Med.

[13] Kienle GS (2012). Fever in Cancer Treatment: Coley’s Therapy and Epidemiologic Observations. Glob Adv Health Med.

[14] Wiemann B, Starnes CO (1994). Coley’s toxins, tumour necrosis factor and cancer research: a historical perspective. Pharmacol Ther.

[15] Johnston BJ (1962). Clinical effects of Coley’s toxin. I. A controlled study. Cancer Chemother Rep.

[16] Johnston BJ, Novales ET (1962). Clinical effect of Coley’s toxin. II. A seven-year study. Cancer Chemother Rep.

[17] Tang ZY, Zhou HY, Zhao G, Chai LM, Zhou M, Lu JZ, Liu KD, Havas HF, Nauts HC (1991). Preliminary result of mixed bacterial vaccine as adjuvant treatment of hepatocellular carcinoma. Med Oncol Tumor Pharmacother.

[18] Starnes CO (1992). Coley’s toxins in perspective. Nature.

[19] Isenberg J, Stoffel B, Wolters U, Beuth J, Stützer H, Ko HL, Pichlmaier H (1995). Immunostimulation by propionibacteria – effects on immune status and antineoplastic treatment. Anticancer Res.

[20] Richardson MA, Ramirez T, Russell NC, Moye LA (1999). Coley toxins immunotherapy: a retrospective review. Altern Ther Health Med.

[21] McCarthy EF (2006). The toxins of William B. Coley and the treatment of bone and soft-tissue sarcomas. Iowa Orthop J.

[22] Tsung K1, Norton JA (2006). Lessons from Coley’s Toxin. Surg Oncol.

[23] Nagorsen D, Marincola FM, Kaiser HE (2002). Bacteria-related spontaneous and therapeutic remission of human malignancies. In Vivo.

[24] Takita H (1970). Effect of postoperative empyema on survival of patients with bronchogenic carcinoma. J Thorac Cardiovasc Surg.

[25] Ruckdeschel JC, Codish SD, Stranahan A, McKneally MF (1972). Postoperative empyema improves survival in lung cancer. Documentation and analysis of a natural experiment. N Engl J Med 19.

[26] Cady B, Cliffton EE (1967). Empyema and survival following surgery for bronchogenic carcinoma. J Thorac Cardiovasc Surg.

[27] Brohee D, Vanderhoeft P, Smets P (1977). Lung cancer and postoperative empyema. Eur J Cancer.

[28] Minasian H, Lewis CT, Evans SJ (1978). Influence of postoperative empyema on survival after pulmonary resection for bronchogenic carcinoma. Br Med J.

[29] Müller W, Regazzoni P (1975). Does a local postoperative infection improve the prognosis in colonic carcinoma [Article in German]. Helv Chir Acta.

[30] Jackson RM, Rice DH (1990). Wound infections and recurrence in head and neck cancer. Otolaryngol Head Neck Surg.

[31] Grandis JR, Snyderman CH, Johnson JT, Yu VL, D’Amico F (1992). Postoperative wound infection. A poor prognostic sign for patients with head and neck cancer. Cancer.

[32] Teucher G, Schindler AE (1987). Postoperative fever and prognosis in breast cancer [Article in German]. Arch Geschwulstforsch.

[33] Papachristou DN, Fortner JG (1979). Effect of postoperative wound infection on the course of stage II melanoma. Cancer.

[34] Jeys LM, Grimer RJ, Carter SR, Tillman RM, Abudu A (2007). Post operative infection and increased survival in osteosarcoma patients: are they associated?. Ann Surg Oncol.

[35] Dunn GP, Old LJ, Schreiber RD (2004). The three Es of cancer immunoediting. Annu Rev Immunol.

[36] Gerlinger M, Rowan AJ, Horswell S, Larkin J, Endesfelder D, Gronroos E, Martinez P, Matthews N, Stewart A, Tarpey P, Varela I, Phillimore B, Begum S, McDonald NQ, Butler A, Jones D, Raine K, Latimer C, Santos CR, Nohadani M, Eklund AC, Spencer-Dene B, Clark G, Pickering L, Stamp G, Gore M, Szallasi Z, Downward J, Futreal PA, Swanton C (2012). Intratumor heterogeneity and branched evolution revealed by multiregion sequencing. N Engl J Med.

[37] Mantovani A, Allavena P, Sica A, Balkwill B (2008). Cancer-related inflammation. Nature.

[38] Dvorak HF (1986). Tumors: wounds that do not heal. Similarities between tumor stroma generation and wound healing. N Engl J Med.

[39] Haddow A (1972). Molecular repair, wound healing, and carcinogenesis: tumor production a possible overhealing?. Adv Cancer Res.

[40] Ostrand-Rosenberg S, Sinha P (2009). Myeloid-derived suppressor cells: Linking inflammation and cancer. J Immunol.

[41] Rook GA, Dalgleish A (2011). Infection, immunoregulation, and cancer. Immunol Rev.

[42] de Martel C, Ferlay J, Franceschi S, Vignat J, Bray F, Forman D, Plummer M (2012). Global burden of cancers attributable to infections in 2008: a review and synthetic analysis. Lancet Oncol.

[43] Hoption Cann SA, van Netten JP, van Netten C (2006). Acute infections as a means of cancer prevention: opposing effects to chronic infections?. Cancer Det Prev.

[44] Urayama KY, Buffler PA, Gallagher ER, Ayoob JM, Ma X (2010). A meta-analysis of the association between day-care attendance and childhood acute lymphoblastic leukaemia. Int J Epidemiol.

[45] O’Rorke MA, Black C, Murray LJ, Cardwell CR, Gavin AT, Cantwell MM (2013). Do perinatal and early life exposures influence the risk of malignant melanoma? A Northern Ireland birth cohort analysis. Eur J Cancer.

[46] van Steensel-Moll HA, Valkenburg HA, van Zanen GE (1986). Childhood leukemia and infectious diseases in the first year of life: a register-based case–control study. Am J Epidemiol.

[47] Parodi S, Crosignani P, Miligi L, Nanni O, Ramazzotti V, Rodella S, Costantini AS, Tumino R, Vindigni C, Vineis P, Stagnaro E (2013). Childhood infectious diseases and risk of leukaemia in an adult population. Int J Cancer.

[48] Mastrangelo G, Grange JM, Fadda E, Fedeli U, Buja A, Lange JH (2005). Lung cancer risk: effect of dairy farming and the consequence of removing that occupational exposure. Am J Epidemiol.

[49] Laakkonen A, Pukkala E (2008). Cancer incidence among Finnish farmers, 1995–2005. Scand J Work Environ Health.

[50] Portengen L, Sim M, Wouters IM, Heederik D, Vermeulen R (2010). Endotoxin exposure and lung cancer risk: a systematic review and meta-analysis of the published literature on agriculture and cotton textile workers. Cancer Causes Control.

[51] Mastrangelo G, Fadda E, Cegolon L (2013). Endotoxin and cancer chemo-prevention. Cancer Epidemiol.

[52] Enterline PE, Sykora JL, Keleti G, Lange JH (1985). Endotoxins, cotton dust, and cancer. Lancet.

[53] Bray F, Ren JS, Masuyer E, Ferlay J (2013). Global estimates of cancer prevalence for 27 sites in the adult population in 2008. Int J Cancer.

[54] de Vries ED, Coebergh J (2004). Cutaneous malignant melanoma in Europe. Eur J Cancer.

[55] Linos E, Swetter S, Cockburn M, Colditz G, Clarke C (2009). Increasing burden of melanoma in the United States. J Invest Dermatol.

[56] Hoffman FL (1916). The mortality from cancer in the Western hemisphere. J Cancer Res.

[57] Bach J-F (2002). The effect of infections on susceptibility to autoimmune and allergic diseases. N Engl J Med.

[58] Bottasso O, Docena G, Stanford JL, Grange JM (2009). Chronic inflammation as a manifestation of defects in immunoregulatory networks – implications for novel therapies based on microbial products. Inflammopharmacol.

[59] Krone B, Grange JM (2010). Melanoma, Darwinian medicine and the inner world. J Cancer Res Clin Oncol.

[60] Cho I, Blaser MJ (2012). The human microbiome: at the interface of health and disease. Nat Rev Genet.

[61] Weinstock GM (2012). Genomic approaches to studying the human microbiota. Nature.

[62] Rook GA (2009). The hygiene hypothesis and Darwinian Medicine.

[63] Rook GA (2010). 99th Dahlem conference on infection, inflammation and chronic inflammatory disorders: Darwinian medicine and the ‘hygiene’ or ‘old friends’ hypothesis. Clin Exp Immunol.

[64] Oliveira EC1, Leite MS, Miranda JA, Andrade AL, Garcia SB, Luquetti AO, Moreira H (2001). Chronic Trypanosoma cruzi infection associated with low incidence of 1,2-dimethylhydrazine-induced colon cancer in rats. Carcinogenesis.

[65] Hunter CA1, Yu D, Gee M, Ngo CV, Sevignani C, Goldschmidt M, Golovkina TV, Evans S, Lee WF, Thomas-Tikhonenko A (2001). Cutting edge: systemic inhibition of angiogenesis underlies resistance to tumors during acute toxoplasmosis. J Immunol.

[66] Hibbs JB, Lambert LH, Remington JS (1971). Resistance to murine tumors conferred by chronic infection with intracellular protozoa, Toxoplasma gondii and Besnoitia jellisoni. J Infect Dis.

[67] de St GF, Webster RG (1966). Disquisitions of Original Antigenic Sin. I. Evidence in man. J Exp Med.

[68] Krone B, Oeffner F, Grange JM (2009). Is the risk of multiple sclerosis related to the ‘biography’ of the immune system?. J Neurol.

[69] Holmgren I (1935). La tuberculine di le BCG chez les concereux. Schweiz Med Wochenschr.

[70] Tan JK, Ho VC (1993). Pooled analysis of the efficacy of bacilli Calmette–Guérin (BCG) immunotherapy in malignant melanoma. J Dermatol Surg Oncol.

[71] Grange JM, Stanford JL (1990). BCG vaccination and cancer. Tubercle.

[72] Häro AS (1986). The effect of BCG-vaccination and tuberculosis on the risk of leukaemia. Dev Biol Stand.

[73] Brown WM, Doll R (1961). Leukaemia in childhood and young adult life. Brit Med J.

[74] Grange JM, Stanford JL (1994). Aetiology of childhood leukemia. Arch Dis Child.

[75] Colditz GA, Brewer TF, Berkey CS, Wilson ME, Burdick E, Fineberg HV, Mosteller F (1994). Efficacy of BCG vaccine in the prevention of tuberculosis. Meta-analysis of the published literature. J Amer Med Assoc.

[76] Fine PEM (1995). Variation in protection by BCG: implications of and for heterologous immunity. Lancet.

[77] Lalor MK, Floyd S, Gorak-Stolinska P, Ben-Smith A, Weir RE, Smith SG, Newport MJ, Blitz R, Mvula H, Branson K, McGrath N, Crampin AC, Fine PE, Dockrell HM (2011). BCG vaccination induces different cytokine profiles following infant BCG vaccination in the UK and Malawi. J Infect Dis.

[78] Berwick M, Anja B (2011). Melanoma epidemiology. Melanoma Development. Molecular Biology Genetics and clinical application.

[79] Kölmel KF, Gefeller O, Haferkamp B (1992). Febrile infections and malignant melanoma: results of a case control study. Melanoma Res.

[80] Kölmel KF, Pfahlberg A, Mastrangelo G, Niin M, Botev IN, Seebacher C, Schneider D, Lambert D, Shafir R, Kokoschka EM, Kleeberg UR, Henz BM, Gefeller O (1999). Infections and melanoma risk: results of a multicentre EORTC case–control study. Melanoma Res.

[81] Pfahlberg A, Kölmel KF, Grange JM, Mastrangelo G, Krone B, Botev IN, Niin M, Seebacher C, Lambert D, Shafir R, Schneider D, Kokoschka EM, Kleeberg UR, Uter W, Gefeller O (2002). Inverse association between melanoma and previous vaccinations against tuberculosis and smallpox: results of the FEBIM study. J Invest Dermatol.

[82] Krone B, Kölmel KF, Grange JM, Mastrangelo G, Henz BM, Botev IN, Niin M, Seebacher C, Lambert D, Shafir R, Kokoschka EM, Kleeberg UR, Gefeller O, Pfahlberg A (2003). Impact of vaccinations and infectious diseases on the risk of melanoma - evaluation of an EORTC case–control study. Eur J Cancer.

[83] Krone B, Kölmel KF, Henz BM, Grange JM (2005). Protection against melanoma by vaccination with Bacille Calmette-Guérin (BCG) and/or vaccinia: an epidemiology-based hypothesis on the nature of a melanoma risk factor and its immunological control. Eur J Cancer.

[84] Schiavetti F, Thonnard J, Colau D, Boon T, Coulie PG (2002). A human endogenous retroviral sequence encoding an antigen recognized on melanoma by cytolytic T lymphocytes. Cancer Res.

[85] Serafino A, Balestrieri E, Pierimarchi P, Matteucci C, Moroni G, Oricchio E, Rasi G, Mastino A, Spadafora C, Garaci E, Vallebona PS (2009). The activation of human endogenous retrovirus K (HERV-K) is implicated in melanoma cell malignant transformation. Exp Cell Res.

[86] Krone B, Grange JM (2013). Is a hypothetical melanoma-like neuromelanin the underlying factor essential for the aetiopathogenesis and clinical manifestations of multiple sclerosis?. BMC Neurol.

[87] Krone B, Hunsmann G, Georg August University Göttingen, inventors (2011). Preventive vaccination against melanoma.

[88] Muster T, Waltenberger A, Grassauer A, Hirschl S, Caucig P, Romirer I, Födinger D, Seppele H, Schanab O, Magin-Lachmann C, Löwer R, Jansen B, Pehamberger H, Wolff K (2003). An endogenous retrovirus derived from human melanoma cells. Cancer Res.

[89] Depil S, Roche C, Dussart P, Prin L (2002). Expression of a human endogenous retrovirus, HRV-K, in the blood cells of leukaemia patients. Leukaemia.

[90] Contreras-Galindo R, Kaplan MH, Leissner P, Verjat T, Ferlenghi I, Bagnoli F, Giusti F, Dosik MH, Hayes DF, Gitlin SD, Markovitz DM (2008). Human endogenous retrovirus K (HML-2) elements in the plasma of people with lymphoma and breast cancer. J Virol.

[91] Wang-Johanning F, Radvanyi L, Rycaj K, Plummer JB, Yan P, Sastry KJ, Piyathilake CJ, Hunt KK, Johanning GL (2008). Human endogenous retrovirus K triggers an antigen-specific immune response in breast cancer patients. Cancer Res.

[92] Jordan MS, Boestanu A, Reed AJ, Petrone AL, Holenbeck AE, Lerman MA, Naji A, Caton AJ (2001). Thymic selection of CD4 + CD25+ regulatory T cells induced by an agonistic self-peptide. Nat Immunol.

[93] Dhanji S, Teh HS (2003). IL-2-Activated CD8 + CD44high cells express both adaptive and innate immune system receptors and demonstrate specificity for syngeneic tumour cells. J Immunol.

[94] Mathis D, Benoist C (2009). Aire. Annu Rev Immunol.

[95] Kershaw MH, Hsu C, Mondesire W, Parker LL, Wang G, Overwijk WW, Lapointe R, Yang JC, Wang RF, Restifo NP, Hwu P (2001). Immunization against endogenous retroviral tumor-associated antigens. Cancer Res.

[96] Grange JM, Krone B, Kölmel K, Mastrangelo G (2009). Editorial: Can prior vaccinations against certain infections confer protection against developing melanoma?. Med J Aust.

[97] Dissanayake D, Gronski MA, Lin A, Elford AR, Ohashi PS (2010). Immunological perspective of self versus tumor antigens: insights from the RIP-gp model. Immunol Rev.

[98] Lin AC, Dissanayake D, Dhanji S, Elford AR, Ohashi PS (2011). Different toll-like receptor stimuli have a profound impact on cytokines required to break tolerance and induce autoimmunity. PLoS One.

[99] Krone B, Grange JM (2010). Multiple sclerosis - are protective immune mechanisms compromised by a complex infectious background?. Autoimmune Dis.

[100] Galon J, Mlecnik B, Bindea G, Angell HK, Berger A, Lagorce C, Lugli A, Zlobec I, Hartmann A, Bifulco C, Nagtegaal ID, Palmqvist R, Masucci GV, Botti G, Tatangelo F, Delrio P, Maio M, Laghi L, Grizzi F, Asslaber M, D'Arrigo C, Vidal-Vanaclocha F, Zavadova E, Chouchane L, Ohashi PS, Hafezi-Bakhtiari S, Wouters BG, Roehrl M, Nguyen L, Kawakami Y (2014). Towards the introduction of the ‘Immunoscore’ in the classification of malignant tumours. J Pathol.

[101] Schaade L, Kleines M, Walter R, Thomssen R, Ritter K (1999). A membrane-located glycosphingolipid of monocyte/granulocyte lineage cells induces growth arrest and triggers the lytic viral cycle in Epstein-Barr virus genome-positive Burkitt lymphoma lines. Med Microbiol Immunol.

[102] Schaade L, Kleines M, Krone B, Hausding M, Walter R, Ritter K (2000). Enhanced transcription of the s-adenosylhomocysteine hydrolase gene precedes Epstein-Barr virus lytic gene activation in ganglioside-stimulated lymphoma cells. Med Microbiol Immunol.

[103] Maas D, Maret C, Schaade L, Scheithauer S, Ritter K, Kleines M (2006). Reactivation of the Epstein-Barr virus from viral latency by an S-adenosylhomocysteine hydrolase/14-3-3 zeta/PLA2-dependent pathway. Med Microbiol Immunol.

[104] Tran T, Burt D, Eapen L, Keller OR (2013). Spontaneous regression of metastatic melanoma after inoculation with tetanus-diphtheria-pertussis vaccine. Curr Oncol.

[105] Mastrangelo G, Krone B, Fadda E, Buja A, Grange JM, Rausa G, de Vries E, Koelmel KF (2009). Does yellow fever 17D vaccine protect against melanoma?. Vaccine.

[106] Rimpelä AH, Pukkala EI (1987). Cancers of affluence: positive social class gradient and rising incidence trend in some cancer forms. Soc Sci Med.

[107] Mastrangelo G, Cegolon L (2012). Padova: Personal communication.

[108] Hodges-Vazquez M, Wilson JP, Hughes H, Garman P (2011). The yellow fever 17D vaccine and risk of malignant melanoma in the United States military. Vaccine.

[109] Kölmel KF, Grange JM, Krone B, Mastrangelo G, Rossi CR, Henz BM, Seebacher C, Botev IN, Niin M, Lambert D, Shafir R, Kokoschka EM, Kleeberg UR, Gefeller O, Pfahlberg A (2005). Prior immunisation of patients with malignant melanoma with vaccinia or BCG is associated with better survival. An European Organization for Research and Treatment of Cancer cohort study on 542 patients. Eur J Cancer.

[110] Huang G, Li Z, Wan X, Wang Y, Dong J (2013). Human endogenous retroviral K element encodes fusogenic activity in melanoma cells. J Carcinog.

[111] Cegolon L, Salata C, Weiderpass E, Vineis P, Palù G, Mastrangelo G (2013). Human endogenous retroviruses and cancer prevention: evidence and prospects. BMC Cancer.

[112] Sedgwick P (2014). Nested case–control studies: advantages and disadvantages. BMJ.

[113] Gaucher D, Therrien R, Kettaf N, Angermann BR, Boucher G, Filali-Mouhim A, Moser JM, Mehta RS, Drake DR, Castro E, Akondy R, Rinfret A, Yassine-Diab B, Said EA, Chouikh Y, Cameron MJ, Clum R, Kelvin D, Somogyi R, Greller LD, Balderas RS, Wilkinson P, Pantaleo G, Tartaglia J, Haddad EK, Sékaly RP (2008). Yellow fever vaccine induces integrated multilineage and polyfunctional immune responses. J Exp Med.

[114] Pulendran B (2009). Learning immunology from the yellow fever vaccine: innate immunity to systems vaccinology. Nat Rev Immunol.

[115] Downey RF, Sullivan FJ, Wang-Johanning F, Ambs S, Giles FJ, Glynn SA (2014). Human endogenous retrovirus K and cancer: Innocent bystander or tumorigenic accomplice?. Int J Cancer.

[CR116] The pre-publication history for this paper can be accessed here:http://www.biomedcentral.com/1471-2407/14/595/prepub

