# Supplementary figures and images for: The biography of the immune system and the control of cancer: from St Peregrine to contemporary vaccination strategies
Source: BMC Cancer. 2014 Aug 16;14:595. doi: 10.1186/1471-2407-14-595 (PMC4141110; doi:10.1186/1471-2407-14-595)

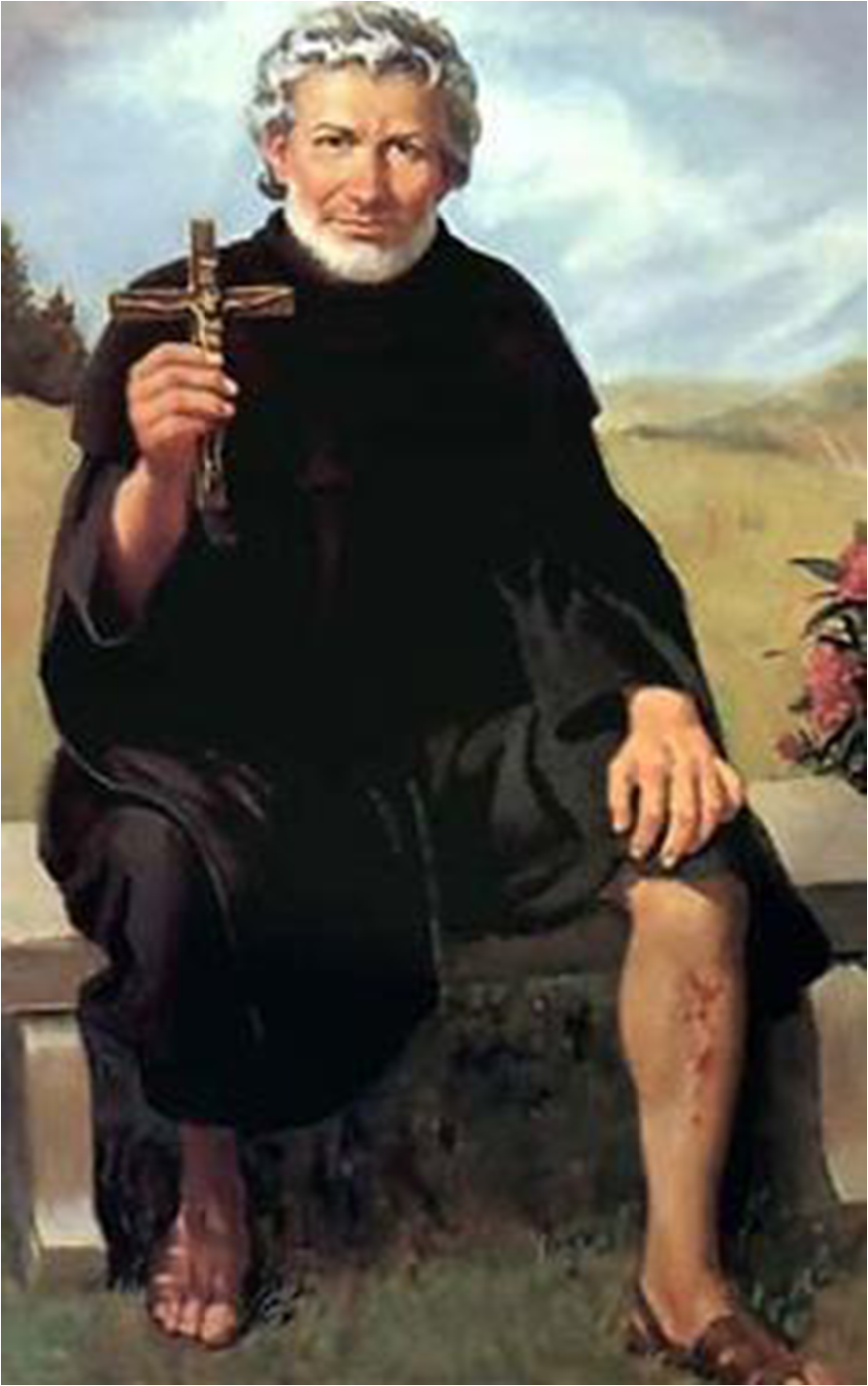

Supplement: Supplementary file 1 — Authors’ original file for figure 1 [file 12885_2013_4777_MOESM1_ESM.tif]

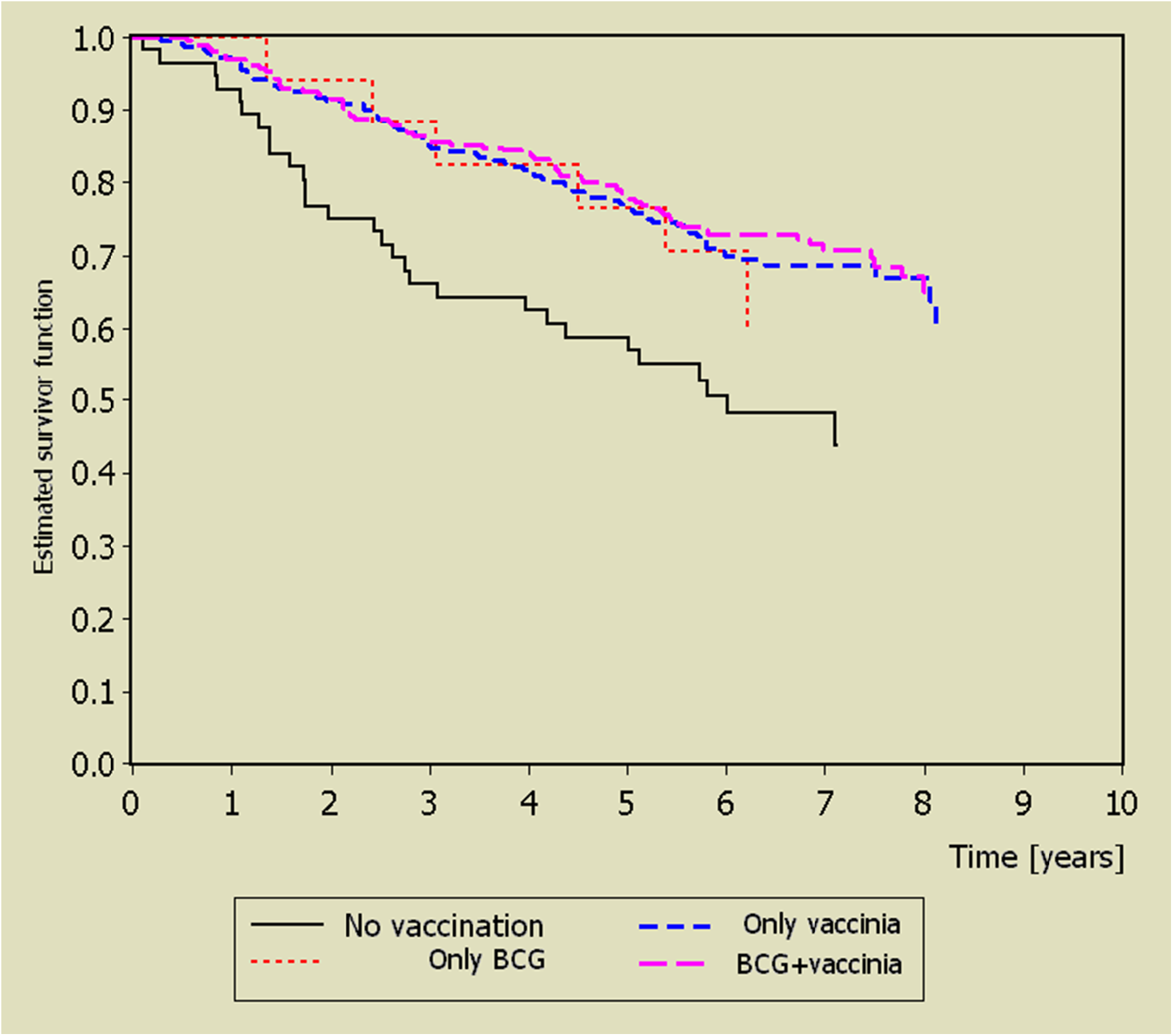

Supplement: Supplementary file 2 — Authors’ original file for figure 2 [file 12885_2013_4777_MOESM2_ESM.tif]
